# Multichannel Retinal Blood Vessel Segmentation Based on the Combination of Matched Filter and U-Net Network

**DOI:** 10.1155/2021/5561125

**Published:** 2021-05-25

**Authors:** Yuliang Ma, Zhenbin Zhu, Zhekang Dong, Tao Shen, Mingxu Sun, Wanzeng Kong

**Affiliations:** ^1^Institute of Intelligent Control and Robotics, Hangzhou Dianzi University, Hangzhou, 310018 Zhejiang, China; ^2^School of Electronics and Information, Hangzhou Dianzi University, Hangzhou, 310018 Zhejiang, China; ^3^School of Electrical Engineering, University of Jinan, Jinan, 250022 Shandong, China; ^4^Key Laboratory of Brain Machine Collaborative Intelligence of Zhejiang Province, Hangzhou, 310018 Zhejiang, China

## Abstract

Aiming at the current problem of insufficient extraction of small retinal blood vessels, we propose a retinal blood vessel segmentation algorithm that combines supervised learning and unsupervised learning algorithms. In this study, we use a multiscale matched filter with vessel enhancement capability and a U-Net model with a coding and decoding network structure. Three channels are used to extract vessel features separately, and finally, the segmentation results of the three channels are merged. The algorithm proposed in this paper has been verified and evaluated on the DRIVE, STARE, and CHASE_DB1 datasets. The experimental results show that the proposed algorithm can segment small blood vessels better than most other methods. We conclude that our algorithm has reached 0.8745, 0.8903, and 0.8916 on the three datasets in the sensitivity metric, respectively, which is nearly 0.1 higher than other existing methods.

## 1. Introduction

The human eyes consist of the following parts: cornea, pupil, iris, vitreous, and retina. Abnormalities in any of these tissue structures may cause vision defects or even blindness. Among them, the study of retinal structure and its blood vessels is significant [1]. The extraction of retinal blood vessels and the characterization of morphological properties, such as diameter, shape, distortion, and bifurcation, can be used to screen, evaluate, and treat different ocular abnormalities [2]. Evaluation of retinal vascular properties, such as changes in width, is used to analyze hypertension, while bifurcation points and tortuosity can help identify cardiovascular disease and diabetic retinopathy [3].

The retinal vessel extraction methods, including pattern recognition, are classified into five core classes [4]. The pattern recognition techniques are generally divided into two categories: supervised learning and unsupervised learning. The supervised learning method needs to use manual segmentation images of ophthalmologists for training. This method requires many training images, and the training time is longer than that of other methods, but this method has an excellent generalized effect and can be applied to other images of the same type. Compared with supervised learning, nonsupervised learning methods, such as matched filtering, mathematical morphology operations, blood vessel tracking, and clustering, do not require corresponding image labels but analyze and process based on the existing data. These two types of methods have been applied and innovated by many researchers in recent years.

### 1.1. Unsupervised Learning Methods

Literature [5] proposed a new kernel-based technique, viz, Fréchet PDF-based matched filter. The new method performs a better matching between the vessel profile and Fréchet template. Literature [6] improved the extraction method of blood vessels, using a series of morphological operations to extract small blood vessels, and finally fused with the segmented image to supplement the small blood vessels. Compared with other algorithms, it can segment as many tiny blood vessels as possible. However, the steps of the algorithm are too complicated, and although the final segmentation effect obtains the smallest blood vessels, the small blood vessels are in an intermittent state as a whole, and they are not well connected with thicker blood vessels. Literature [7] proposed a new matched filtering method, which applies contrast-limited adaptive histogram equalization and Gaussian second-derivative-based matched filter in preprocessing and uses an entropy-based optimal threshold method performing binarization. This algorithm effectively improves the sensitivity metric of segmentation, but like literature [6], it does not perform well with accuracy. Literature [8] proposed an automatic segmentation method of retinal blood vessels using a matched filter and fuzzy *C*-means clustering. The algorithm uses contrast-limited adaptive histogram equalization to enhance the contrast of the image. After using Gabor and Frangi filters for noise removal and background removal, the fuzzy *C*-means are used to extract the initial vascular network, and the integrated level set method is used to refine segmentation further. The algorithm has good sensitivity and specificity. The problem is that the ability to segment small blood vessels is limited, and many segmentation details are missed. Literature [9] proposed a novel method to extract the retinal blood vessel using local contrast normalization and a second-order detector. The proposed methodology achieves higher accuracy in vessel segmentation than existing techniques. Literature [10] proposed a novel matched filter approach with the Gumbel probability distribution function as its kernel. The reason to achieve the higher accuracy is due to a better matching filter with the Gumbel PDF-based kernel.

### 1.2. Supervised Learning Methods

Literature [11] proposed a method using deep conventional neural networks and a hysteresis threshold method to detect the vessels accurately. The proposed method gives good performance in which more tiny vessels are detected. Literature [12] proposed a multilevel CNN model applied for automatic blood vessel segmentation in retinal fundus images. A novel max-resizing technique is proposed to improve the generalization of the training procedure for predicting blood vessels from retinal fundus images. Literature [13] proposed a new segment-level loss used with the pixel-wise loss to balance the importance between thick vessels and thin vessels in the training process. Literature [14] proposed a cross-connected convolutional neural network (CcNet) to automatically segment retinal vessel trees. The cross connections between a primary path and a secondary path fuse the multilevel features. This method has relatively advanced performances, including competitive strong robustness and segmentation speed. Literature [15] proposed a method for retinal vessel segmentation using patch-based fully convolutional networks. Literature [16] applied dilated convolutions in a deep neural network to improve the segmentation of retinal blood vessels from fundus images. Literature [17] proposed a new improved algorithm based on the U-Net network model. The algorithm integrates the Inception-Res structure module and the Dense-Inception structure module into the U-Net structure. The algorithm dramatically deepens the depth of the network but does not add additional training parameters. It has good segmentation performance in the image segmentation of retinal blood vessels and has strong generalization ability. Literature [18] proposed a new hybrid algorithm for retinal vessel segmentation on fundus images. The proposed algorithm applies a new directionally sensitive blood vessel enhancement before sending fundus images to U-Net. Literature [19] proposed a supervised method based on a pretrained fully convolutional network through transfer learning. This method simplifies the typical retinal vessel segmentation problem into regional semantic vessel element segmentation tasks. Generally, unsupervised methods are less complex and suffer from relatively lower accuracy than supervised methods [13].

To solve the problem of insufficient segmentation of small blood vessels in most papers, we have devised a new automatic segmentation framework for retinal vessels based on improving U-Net and a multiscale matched filter. The creative points of this paper are summarized as follows:

1. We proposed an improved black hat algorithm to enhance the characteristics of blood vessels and reduce the interference of other tissues
2. An algorithm combining a multiscale matched filter and U-Net neural network is proposed. This paper mainly uses the improved U-Net convolutional neural network combined with a multiscale matched filter to perform multichannel blood vessel segmentation processing on the retinal fundus image
3. We have devised a new loss function to train the improved U-Net neural network to solve pixel imbalance in the image better

The rest of this paper is organized as follows.  Section 2 outlines the proposed method and datasets. The performance of the proposed method and the discussion are described in detail in Section 3. A conclusion is drawn in Section 4.

## 2. Materials and Methods

### 2.1. System Overview

**Figure 1:**
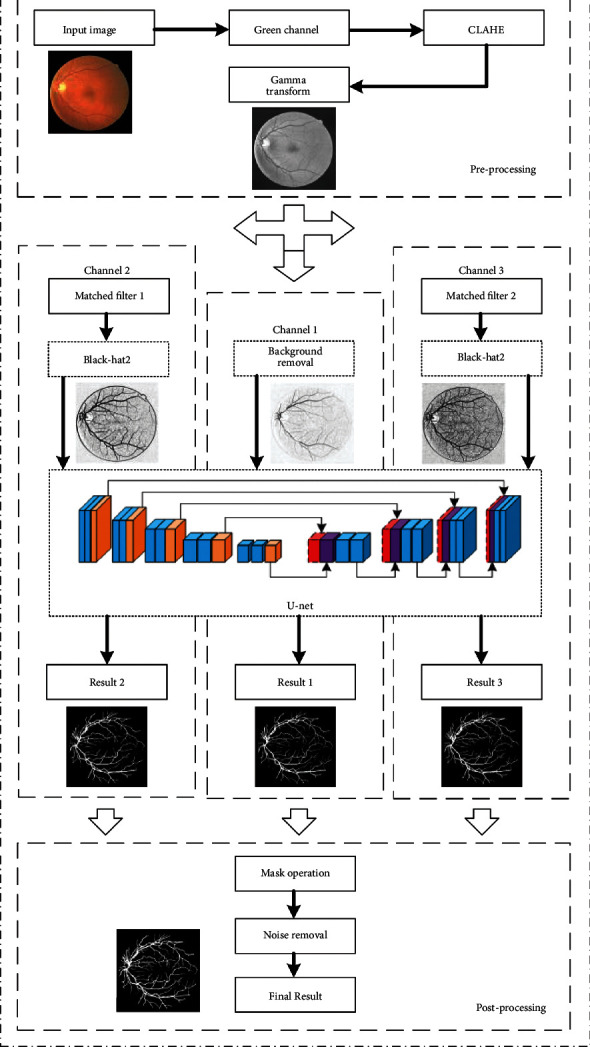
Proposed model.

The proposed algorithm consists of three steps: preprocessing datasets, training U-Net in 3 channels, and postprocessing. This algorithm's main feature extraction framework is based on the improved U-Net model, using three feature extraction channels. It is mainly to perform a whole feature extraction of the image in channel 1 so that some morphological operations are performed in the preprocessing part to reduce image artifacts and noise. On the remaining two channels, matched filters are used to extract retinal vessels of different scales, and then, the improved U-Net model is used to extract features, and the OR-type operator is used to fuse the final output image. Experimental results verify that the image processed by multichannel matched filtering is better than the unprocessed image. The overall flowchart is shown in Figure 1.

### 2.2. Datasets

To verify the effectiveness of the algorithm in this paper, this paper chooses three commonly used public datasets for training and testing: DRIVE, STARE, and CHASE_DB1 datasets. These datasets include a wide range of challenging images. The DRIVE contains 40 color retinal fundus images divided into a training set and a testing set. The plane resolution of DRIVE is 565 × 584. The STARE contains 20 color retinal fundus images with a resolution of 605 × 700 pixels. Unlike the DRIVE, this dataset does not have a training set and a testing set. The CHASE_DB1 contains 28 color retinal fundus images with a resolution of 960 × 999 pixels, and the training set and testing set are also not divided. Each image in these three datasets has a label of retinal blood vessel image segmented manually by two professional physicians. We randomly selected 5 images in the STARE dataset as test images (im0002, im0077, im0163, im0255, and im0291), and the remaining 15 images were set as the training set. In CHASE_DB1, we select the last 8 images as the test set and the remaining 20 images as the training set. Note that mask images of STARE and CHASE_DB1 are not available, so we extracted the green channel of the images and then used some morphological algorithms and threshold algorithm to obtain the mask images.

### 2.3. Preprocessing

**Figure 2:**
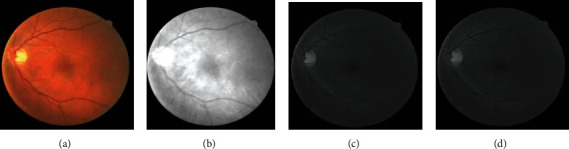
Color fundus image and its different RGB channels: (a) original RGB image; (b) red channel; (c) green channel; (d) blue channel.

In this paper, the green channel is selected as the input image of the preprocessing part. This is because the retinal blood vessels presented by the green channel have better contrast with the background compared with the red channel and the blue channel [20, 21], as shown in Figure 2.

**Figure 3:**
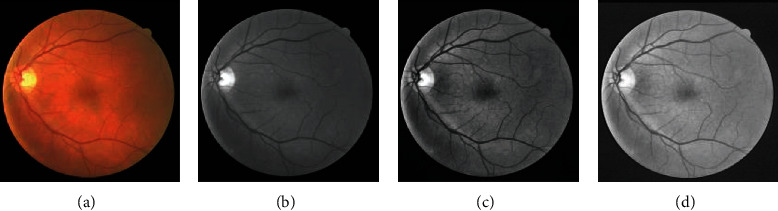
Typical images after each preprocessing step: (a) original RGB image; (b) red channel; (c) image after CLAHE operation; (d) image after gamma correction.

It can be seen from Figure 2 that the appearance of blood vessels on the green channel of the color image consists of more information compared to that on the red and blue channel images, but the overall image is still dark, and the contrast is not obvious. In order to improve this situation, adaptive histogram threshold processing (CLAHE) [22] and gamma transformation are performed on the extracted green channel grayscale image, as shown in Figure 3. In this part of the process, CLAHE is used to enhance the contrast between the nonvessels and blood vessels, and gamma transformation is used to adjust and reduce the background noise in the image. We can see Tables 1–3 in Supplementary Materials for a comprehensive comparison of blood vessel enhanced algorithms, and these data can prove that the CLAHE method improves the general performance of the proposed method.

### 2.4. Multichannel Feature Extraction

#### 2.4.1. Channel 1

In order to retain all the blood vessel feature information of the image as much as possible, some morphological operations are used in channel 1 to remove background noise, and then, the U-Net network is used for feature extraction. For the artifacts caused by uneven illumination in the image and nonvascular structures, we use the morphological closing operation algorithm to estimate the background and then perform the result using the mathematical operation shown in equation (1).

**Figure 4:**
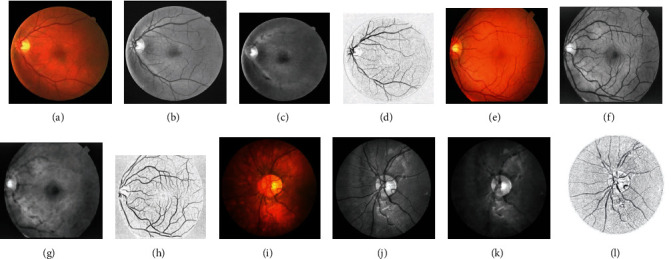
(a, e, i) The fundus images, (b, f, j) green channel images applying CLAHE and gamma transformation, (c, g, k) background extracted by close operation, and (d, h, l) the final results. The different samples of (a–d) DRIVE, (e–h) STARE, and (i–l) CHASE_DB1.

It can be seen intuitively from Figure 4 that the brighter video disc structure in the original image is removed, and most of the artifacts are also processed.

(1) $$\begin{array}{c} \left\{\begin{array}{c} g\left(x,y\right)=255-\left(I_{\text{close}}\left(x,y\right)-I\left(x,y\right)+\frac{1}{m*n}\overset{m}{\underset{x=1}{\sum }}\overset{n}{\underset{y=1}{\sum }}I_{\text{clos}e}\left(x,y\right)\right),\\ f\left(x,y\right)=\frac{255}{\max\left(g\left(x,y\right)\right)-\min\left(g\left(x,y\right)\right)}*\left|g\left(x,y\right)-\min\left(g\left(x,y\right)\right)\right|, \end{array}\right. \end{array}$$

where *f*(*x*, *y*) is the processed image and *I*_close_(*x*, *y*) is the image after a morphological closing operation. We select disk type structuring elements for the closing operator having a radius of eleven pixels. *I*(*x*, *y*) is the original image; *m* and *n* are the image pixel size.

#### 2.4.2. Channel 2

By analyzing the gray image of retinal blood vessels, it can be found that the cross-sectional gray intensity of blood vessels is distributed in an inverted Gaussian curve, the gray value of the center line of the blood vessel is low, and the gray value at the edge of the blood vessel is high [5]. Aiming at this remarkable feature of retinal blood vessel images, Chaudhuri et al. [23] designed a Gaussian matched filter and used its distribution to simulate the grayscale intensity distribution of blood vessel cross sections and filter the blood vessels in sections. In this paper, the matched filters are used in channel 2 and channel 3 to separately enhance and extract the large and small blood vessels to realize the comprehensive segmentation of retinal blood vessels.

Define the two-dimensional Gaussian kernel function as

(2) $$\begin{array}{c} K\left(x,y\right)=-e^{-\left(x^{2}/2s^{2}\right)},\left|y\right|\leq \frac{l}{2}, \end{array}$$

where *s* is the width of the Gaussian kernel and *l* is the length of the Gaussian kernel. The blood vessel starts from the center of the optic disc and extends in multiple directions. Rotating the Gaussian kernel is used to filter the multidirectional blood vessels.

Assuming that *p*(*x*, *y*) is a discrete point in the kernel function, the rotation matrix is

(3) $$\begin{array}{c} g_{i}=\left[\begin{array}{cc} \cos\theta_{i} & -\sin\theta_{i}\\ \sin\theta_{i} & \cos\theta_{i} \end{array}\right]. \end{array}$$

*θ*_*i*_(0 ≤ *θ*_*i*_ ≤ *p*) is the angle of the *i*-th kernel function, and the coordinate value of *p*(*x*, *y*) after rotation is ${\bar{p}}_{i}=\left(u,v\right)$; then, the *i*-th template kernel function is

(4) $$\begin{array}{c} K_{i}\left(x,y\right)=-e^{-\left(u^{2}/2s^{2}\right)},\;\forall {\bar{p}}_{i}\in N, \end{array}$$

where *N* is the template field, and the value range is

(5) $$\begin{array}{c} N=\left\{\left(u,v\right),\left|u\right|\leq 3s,\left|v\right|\leq \frac{l}{2}\right\}. \end{array}$$

In actual algorithm applications, it is often necessary to consider the mean value of the correlation coefficient of the template filter, as shown in

(6) $$\begin{array}{c} m_{i}=\underset{{\bar{p}}_{i}\in N}{\sum }\frac{K_{i}\left(x,y\right)}{A}. \end{array}$$

Among them, *A* represents the number of points in the template area. So, the final template kernel function is

(7) $$\begin{array}{c} K_{i}^{\prime }\left(x,y\right)=K_{i}\left(x,y\right)-m_{i},\;\forall {\bar{p}}_{i}\in N. \end{array}$$

**Figure 5:**
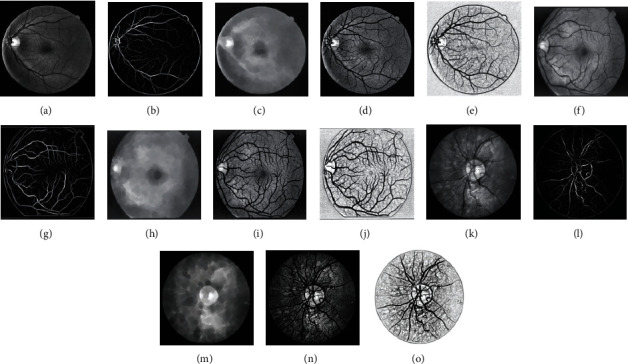
(a, f, k) The grayscale images after preprocessing operation, (b, g, l) large-scale matched filtered images, background extracted by (c, h, m) close operation and (d, i, n) subtraction operation, and (e, j, o) final results. The different samples of (a–e) DRIVE, (f–j) STARE, and (k–o) CHASE_DB1.

This paper improves and optimizes the dependence of Gaussian matched filter response on a vessel diameter. The image enhancement result using large-scale Gaussian matched filtering in channel 2 is shown in Figure 5, where the parameters are set to *l* = 10.8, *s* = 1.9, and 8 directions which means *i* = [1, 2, ⋯, 8] in equation (3). It can be seen from the image that the algorithm has a better segmentation effect for thicker blood vessels and strong antinoise, but it has a poor segmentation effect on small blood vessels, and there is a problem that the smaller blood vessels cannot be distinguished from the background, and the blood vessels are easily broken. In order to solve this problem, this paper proposes an improved method based on the black hat algorithm, which can effectively reduce the influence of background noise by subtracting the original image before matching filter processing and the obtained image after processing to enhance the characteristics of blood vessels. We performed a series of processing transformations as shown in equations (8) and (9) on the images processed by large-scale matched filtering. We call this algorithm black hat2.

(8) $$\begin{array}{c} B_{\text{hat}}\left(f\right)=\left(f\left(x,y\right)∙b\left(u,v\right)\right)-f\left(x,y\right), \end{array}$$

(9) $$\begin{array}{c} g\left(x,y\right)=255-f\left(x,y\right)-2*B_{\text{hat}}\left(f\right), \end{array}$$

where ∙ is the morphological closing operation and *b*(*u*, *v*) is disk type structuring element, *B*_hat_(*f*) is the black hat transformation, *f*(*x*, *y*) is the original image, and *g*(*x*, *y*) is the final processed image.

#### 2.4.3. Channel 3

**Figure 6:**
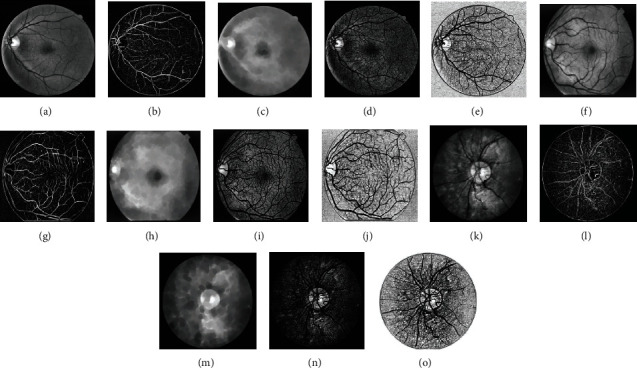
(a, f, k) The grayscale images after preprocessing operation, (b, g, l) small-scale matched filtered images, background extracted by (c, h, m) close operation and (d, i, n) subtraction operation, and (e, j, o) final results. The different samples of (a–e) DRIVE, (f–j) STARE, and (k–o) CHASE_DB1.

This paper uses a small-scale Gaussian matched filter to enhance the image of small blood vessels, as shown in Figure 6. After many experiments, the parameters of the matched filter are set as *l* = 5, *s* = 0.1, and 18 directions which means *i* = [1, 2, ⋯, 18] in equation (3). Using small-scale filters can effectively enhance the small blood vessels in the image, but at the same time, it also enhances much striped noise in the image, and the enhancing effect on the thick blood vessels with central reflection is poor. To reduce the background noise, the black hat2 algorithm used in channel 2 is also used to remove the background in channel 3.

### 2.5. U-Net Model

**Figure 7:**
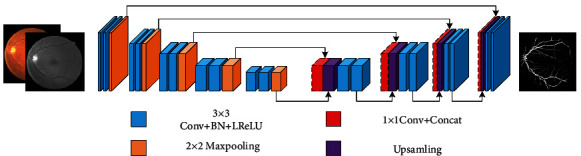
U-Net model architecture.

In image semantic segmentation using deep learning, the U-Net network model is the most widely used, which is improved based on the classic full convolutional network (FCN) [24]. U-Net is an image-to-image pixel-level classification network, and its network structure is apparent, as shown in Figure 7. U-Net is different from other standard segmentation networks: U-Net uses an entirely different feature fusion method—splicing. U-Net stitches the features together in the channel dimension. This method fuses the in-depth features extracted from the image with the shallow features to form thicker features, while the fusion operation of FCN only uses corresponding point addition and does not obtain thicker features.

Unlike the structure in the original literature [24], this paper sets the padding value of 1 in each layer's convolution operation, and the convolution kernel size is 3∗3. The purpose is to ensure that the output and input image sizes are consistent and avoid the size increasing operation in the output layer. It is essentially a binary classification operation in the output layer of U-Net. We use an adaptive threshold segmentation algorithm for processing in this paper. The idea of this algorithm is not to calculate the global image threshold but to calculate the local threshold according to different areas of the image, so for different areas of the image, the algorithm can adaptively calculate different thresholds and perform binary segmentation. The specific calculation process is shown in

(10) $$\begin{array}{c} T=-b+\frac{1}{\left(2m+1\right)\times \left(2n+1\right)}\overset{n}{\underset{i=0}{\sum }}\overset{m}{\underset{j=0}{\sum }}g\left(x\pm i,y\pm j\right), \end{array}$$

where *b* is the fixed parameter, (2 *m* + 1) × (2*n* + 1) is the area, and *T* is the area's threshold.

This paper proposes a new loss function that combines the Dice coefficient with the two-class cross-entropy loss function. The Dice coefficient is widely used in the evaluation of image segmentation. In order to facilitate the formation of the minimized loss function, as shown in

(11) $$\begin{array}{c} L_{\text{dice}}=1-\frac{2\left|X\cap Y\right|}{\left|X\right|+\left|Y\right|}, \end{array}$$

where *X*∩*Y* represents the common elements of the prediction graph and the label graph, *X* and *Y* represent the number of elements of the prediction graph and the label. In order to facilitate the calculation, approximate ∣*X*∩*Y*∣ as the dot product between the predicted probability map and the label, and add the elements in the result. ∣*X*∣ and ∣*Y*∣ are quantified by summing the squares of each element. As shown in

(12) $$\begin{array}{c} L_{\text{dice}}=1-\frac{2\sum_{i}^{N}p\left(k,i\right)q\left(k,i\right)}{\sum_{i}^{N}p^{2}\left(k,i\right)+\sum_{i}^{N}q^{2}\left(k,i\right)}, \end{array}$$

where *N* is the number of pixels, *p*(*k*, *i*) ∈ [0, 1] and *q*(*k*, *i*) ∈ [0, 1] are the predicted probabilities and true labels of the pixel belonging to category *k*.

The cross-entropy loss function used to optimize the network is shown as

(13) $$\begin{array}{c} L_{r}=-\overset{N}{\underset{i}{\sum }}\left[\left(1-\frac{\text{TP}}{N_{\text{p}}}\right)y\log\left(p\right)+\left(1-\frac{\text{TN}}{N_{\text{n}}}\right)\left(1-y\right)\log\left(1-p\right)\right], \end{array}$$

where TP and TN are the numbers of true positive and true negative pixels, respectively; *N*_p_ and *N*_n_ are the numbers of segmented pixels and nonsegmented pixels, respectively; *y* is the label value (*y* = 1, segmentation target; *y* = 0, background); and *p* is the predicted probability value of the pixel.

A coefficient *λ* is introduced to define the new loss function Loss, as shown in

(14) $$\begin{array}{c} \text{Loss}=L_{\text{dice}}+\lambda L_{r}. \end{array}$$

Notably, the coefficient *λ* is set to 0.5 in this work, and the flowchart of U-Net is summarized in Algorithm 1.

### 2.6. Postprocessing

**Figure 8:**
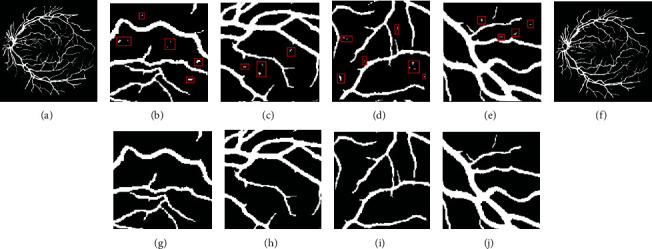
Comparison of postprocessing: (a–e) segmentation image without postprocessing; (f–j) segmentation image applying postprocessing.

In the postprocessing, since the final segmentation image merges the three segmentation images, the noise in the resulting image is also superimposed on all the noises of the three images. Noises will undoubtedly have a significant impact on the actual effect of the segmented image, so this paper addresses this issue in the final postprocessing step. In this paper, a morphological algorithm is used to calculate the size of the connected area of the image. The 8-adjacent connection method is adopted to eliminate the area with the connected area less than 25 pixels, which is to reclassify the area pixels as background. This paper selects a test image in the DRIVE dataset for experimental comparison, and the comparison images are shown in Figure 8.

### 2.7. Experimental Design

#### 2.7.1. U-Net Implementation Details

The U-Net model used in this paper is slightly different from the structure in literature [24]. In order to keep the input and output image sizes of the model consistent, the convolution structure is adjusted accordingly. The specific model structure parameters are shown in Table 1.

In training, we set the epoch to 30 and the initial learning rate lr to 0.01, and then, the learning rate is set to update in a three-stage formula, as shown in

(15) $$\begin{array}{c} \text{lr}=\left\{\begin{array}{cc} 0.01, & \text{epoch}>10,\\ 0.001, & 10<\text{epoch}\leq 20,\\ 0.0001, & 20<\text{epoch}\leq 30. \end{array}\right. \end{array}$$

Setting a larger learning rate at the beginning is to make the model obtain the vicinity of the optimal global parameters faster, and this operation can reduce the training time of the model. After training for a particular epoch, the learning rate needs to be reduced accordingly in order to make the parameters closer to the optimal value in subsequent updates. The stochastic gradient descent (SGD) algorithm is used in the optimization of the loss function.

#### 2.7.2. Training Image Preparation

We randomly select 15 images from STARE and the first 20 images from CHASE_DB1 as their respective training set. Due to the limited number of images in the existing dataset, to avoid the overfitting phenomenon in the model training, we perform data expansion processing on the training set of each dataset. Thanks to the translation invariance of the convolutional structure, the images in the training set in this paper were flipped horizontally and vertically and rotated 180 degrees to increase the amount of data 4 times.

#### 2.7.3. Measuring Metrics

In order to evaluate the segmentation performance of this algorithm, we use the following metrics to perform a comprehensive evaluation of the segmentation result. These metrics are accuracy (ACC), sensitivity (Se), specificity (Sp), and AUC and calculated as follows:

(16) $$\begin{array}{c} \text{ACC}=\frac{\text{TP}+\text{TN}}{\text{TP}+\text{FN}+\text{TN}+\text{FP}}, \end{array}$$

(17) $$\begin{array}{c} \text{Se}=\frac{\text{TP}}{\text{TP}+\text{FN}}, \end{array}$$

(18) $$\begin{array}{c} \text{Sp}=\frac{\text{TN}}{\text{TN}+\text{FP}}, \end{array}$$

(19) $$\begin{array}{c} \text{AUC}=\frac{1}{2}\left(\frac{\text{TP}}{\text{TP}+\text{FN}}+\frac{\text{TN}}{\text{TN}+\text{FP}}\right), \end{array}$$

where TP is true positive, FP is false positive, TN is true negative, and FN is false negative. Se is the sensitivity, which indicates the degree of classification of blood vessels and nonvascular pixels. In this paper, higher sensitivity indicates that more tiny blood vessels can be detected. Sp is specificity, which is used to express the ability of the algorithm to recognize nonvascular pixels. ACC is the accuracy of algorithm segmentation, reflecting the gap between the algorithm segmentation result and the natural result. AUC is the area under the ROC curve, and we adopt another calculation method to get the AUC, as shown in equation (19) [11].

Besides, we also use two other evaluation metrics to measure the effect of segmentation: MCC and CAL.

(20) $$\begin{array}{c} \text{MCC}=\frac{\text{TP}\times \text{TN}-\text{TP}\times \text{FN}}{\sqrt{\left(\text{TP}+\text{FP}\right)\times \left(\text{TP}+\text{FN}\right)\times \left(\text{TN}+\text{FP}\right)\times \left(\text{TN}+\text{FN}\right)}}. \end{array}$$

MCC is a correlation coefficient between the segmentation output of the algorithm and ground truth. It comprehensively considers TP, TN, FP, and FN, which is a relatively balanced metric. Finally, it is more suitable for an imbalanced class ratio.

CAL can be expressed as the product of *C*, *A*, and *L* as follows:

(21) $$\begin{array}{c} f\left(C,A,L\right)=C\times A\times L. \end{array}$$

Suppose *S* and *S*_G_ are the segmentation result and the corresponding ground truth, respectively. These functions are defined as follows:

1. Connectivity (*C*): it evaluates the fragmentation degree between *S* and *S*_*G*_ by comparing the number of connected components:

(22) $$\begin{array}{c} C=1-\min\left(1,\frac{\left|{\#}_{C}\left(S_{\text{G}}\right)-{\#}_{C}\left(S\right)\right|}{\#\left(S_{\text{G}}\right)}\right), \end{array}$$

where #_*C*_(∙) means the number of connected components, while #(∙) means the number of vessel pixels in the considered binary image.

(2) Area (*A*): it evaluates the degree of intersecting area between *S* and *S*_*G*_ and is defined as

(23) $$\begin{array}{c} A=\frac{\#\left(\left(\delta_{\alpha }\left(S\right)\cap S_{G}\right)\cup \left(\delta_{\alpha }\left(S_{G}\right)\cap S\right)\right)}{\#\left(S\cup S_{G}\right)}, \end{array}$$

where *δ*_*α*_(·) is a morphological dilation using a disc of *α* pixels in radius. We set *α* = 2.

(3) Length (*L*): it evaluates the equivalent degree between *S* and *S*_*G*_ by computing the total length:

(24) $$\begin{array}{c} L=\frac{\#\left(\left(\varphi \left(S\right)\cap \delta_{\beta }\left(S_{G}\right)\right)\cup \left(\left(\delta_{\beta }\left(S\right)\cap \varphi \left(S_{G}\right.\right)\right)\right)}{\#\left(\varphi \left(S\right)\cup \varphi \left(S_{G}\right)\right)}, \end{array}$$

where *φ*(·) is the homotopic skeletonization and *δ*_*β*_(∙) is a morphological dilation with a disc of *β* pixel in radius. We set *β* = 2.

According to [26], the CAL metric is essential to quantify thick and thin vessels more equally.

## 3. Results and Discussion

**Figure 9:**
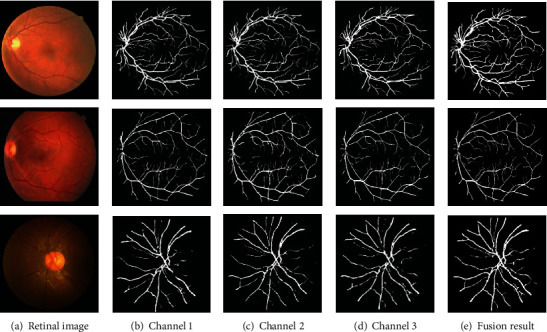
Performance of each channel's segmentation result.

As shown in Figure 9, one test image is selected from each of the three datasets to display the segmentation results of each channel and the fusion results. It can be seen that some of the intermittent blood vessels of each channel are reconnected after fusion, and the number of small blood vessels in the fusion map is significantly higher than that of each channel segmentation map.

The DRIVE dataset is selected as the experimental object and compares the three channels' metric data in this paper. The results show that the overall fusion effect of the three channels is better than the segmentation results of every single channel; in particular, the sensitivity has been dramatically improved, as shown in Table 2.

To illustrate this paper's segmentation effect, we list various metrics on the DRIVE, STARE, and CHASE_DB1 datasets of different papers in recent years in Tables 3–5. It can be seen that the algorithm in this paper is superior to most similar papers in sensitivity and AUC metrics. To have a more comprehensive understanding of the overall segmentation effect of the test set, we show the relevant indicators of the prediction results of all test set images in Table 6. The other essential metrics are MCC and CAL, and they achieved by the proposed method has been contrasted with existing segmentation techniques on the DRIVE, STARE, and CHASE_DB1 datasets shown in Table 7.

**Figure 10:**
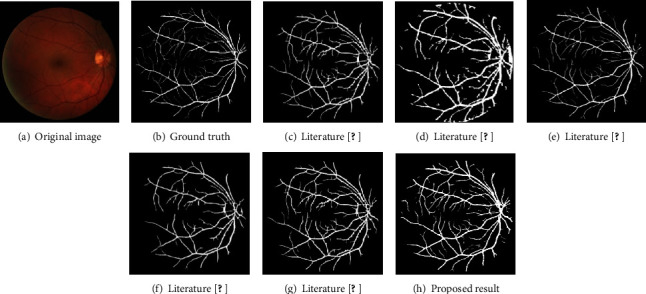
Comparison of different methods on the DRIVE dataset.

We selected image 19_test from the test set of the DRIVE dataset to display the segmentation results, as shown in Figure 10. Literature [5, 27] segmented some small blood vessels, but it is still slightly insufficient compared to this paper's segmentation diagram. Literature [10] lacks many details, and the small blood vessels are not segmented. The segmentation result of literature [11] contains a lot of edge noise, and there are many intermittent blood vessels. Compared with the existing segmentation methods, the segmentation results in this paper have a good performance in terms of the integrity of the whole blood vessels and the segmentation of small blood vessels.

**Figure 11:**
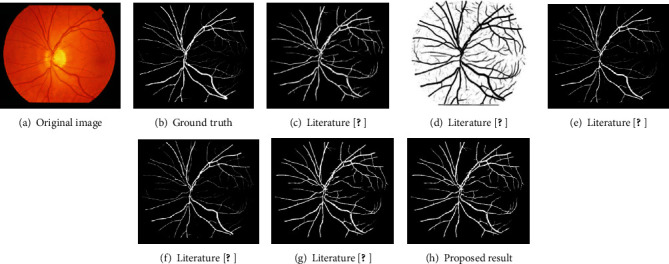
Comparison of different methods on the STARE dataset.

As shown in Figure 11, we select the test results of the image im0163 in the STARE dataset for comparison. It can be shown that the segmentation results of this paper are similar to those of literature [13, 14], but the background noise in literature [13] is not eliminated. Compared with literature [5, 10, 27], the algorithm in this paper illuminates the optic disc structure in the original image as much as possible in the preprocessing part, so the problem that is incorrectly dividing part of the optic disc structure into blood vessels like these papers did not appear in the final segmentation result.

**Figure 12:**
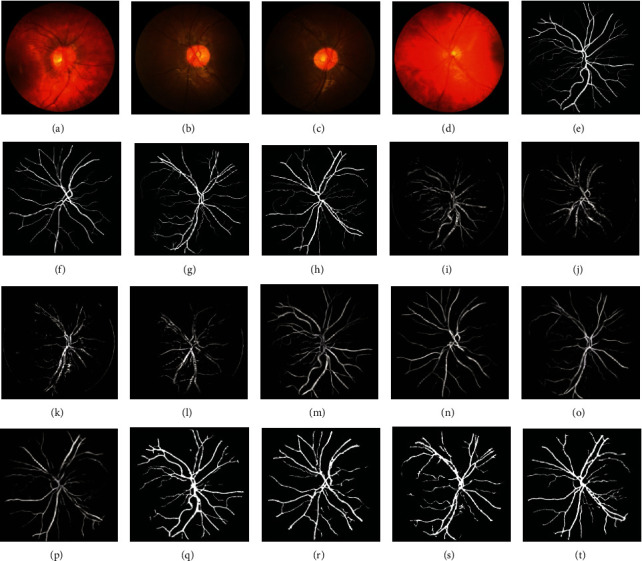
(a, e, i, m, q) Image_12R, (b, f, j, n, r) image_13L, (c, g, k, o, s) image_13R, and (d, h, l, p, t) image_14L from the CHASE_DB1 dataset. (a–d) Original images, (e–h) ground truth, (i–l) literature [19], (m–p) literature [28], and (q–t) proposed segmentation images.

The CHASE_DB1 dataset is not used in most of the papers about retinal blood vessel segmentation. One of the reasons is that the dataset contains half of the abnormal images, which may cause some interference to the trained segmentation model. Meanwhile, this dataset is also a new and challenging dataset compared to the classic DRIVE and STARE datasets. We selected four images image_12R, image_13L, image_13R, and image_14L from the test set of the CHASE_DB1 dataset to compare the segmentation results in order to verify the generalizability of the proposed algorithm, as shown in Figure 12. The segmentation result of the algorithm in literature [19] has much noise, and some blood vessels are not effectively separated. Literature [28] does an excellent job in the segmentation of small blood vessels, but there is a problem that some blood vessels are not connected. Due to the postprocessing in this paper, the segmentation result on this dataset contains less noise and guarantees the continuity of most blood vessels. However, compared with the manual label, some tiny blood vessels cannot be completely segmented from the image background.

The source codes of the proposed framework have been running on the PC (Intel Core i5-6300HQ CPU, 2.30 GHz, 12.0 GB RAM, NVIDIA GTX 950M GPU). DRIVE, STARE, and CHASE_DB1 have spent 11.3 h, 7.1 h, and 16.4 h on training separately in each channel. The average testing time of test images was 1.34 s.  Table 8 shows the parameter comparison of the proposed method with other methods based on U-Net, which can help us compare the framework complexity of different methods. Note that the parameters are not equal to the training time because some methods use slices of a train image as input of the network. For example, literature [19] has 42421 slices as the training set, which means it needs more time to train the network.

## 4. Conclusion

This paper proposes a new retinal blood vessel segmentation method, which combines a multiscale matched filter with a U-Net neural network model of deep learning. First of all, we use an improved morphological image algorithm to effectively reduce the impact of image background in feature extraction. Additionally, in order to avoid ignoring the characteristics of small blood vessels, this paper performs multichannel feature extraction and segmentation on retinal blood vessel images. Finally, the segmented images of the three channels are merged, and various characteristics of retinal blood vessels are obtained as much as possible. In the training of the U-Net model, we used the loss function weighted by the Dice coefficient and the binary cross-entropy to solve the image pixel imbalance problem. The algorithm of this paper is tested on the existing public datasets DRIVE, START, and CHASE_DB1. The experimental results show that there is better performance in four metrics compared with similar papers. The average sensitivity of the algorithm in this paper reached 0.8745, 0.8903, and 0.8916 on the DRIVE, STARE, and CHASE_DB1 datasets, respectively. This result is nearly 0.1 higher than the average sensitivity of other papers. The improvement of the sensitivity metric also reflects that the algorithm in this paper has a good performance in extracting small blood vessels. The focus of this paper is to combine the advantages of unsupervised algorithms and supervised algorithms. We did not make too many improvements to the U-Net network. Therefore, how to prune the deep learning network model structure will be an interesting research direction in the future.

## Figures and Tables

**Algorithm 1:**
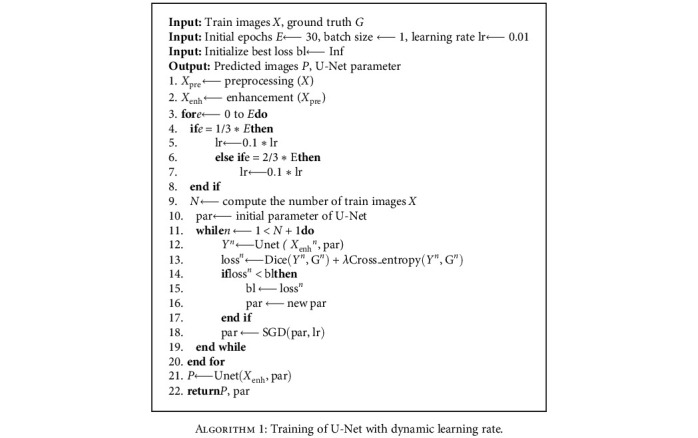
Training of U-Net with dynamic learning rate.

**Table 1:** The parameters of the U-Net architecture.

Block name	Layer name	Image size	Parameters
DoubleConv	Conv (ksize = 3, pad = 1)		(3∗3∗*C*_1_ + 1)∗*C*_2_
	BN + LReLU		2∗*C*_2_
	Conv (ksize = 3, pad = 1)		(3∗3∗*C*_2_ + 1)∗*C*_2_
	BN + LReLU		2∗*C*_2_
			9∗(*C*_1_ + *C*_2_)∗*C*_2_ + 6∗*C*_2_

Input		1 × 576 × 576	0

Encoder block_1	DoubleConv_1	64 × 576 × 576	9∗(1 + 64)∗64 + 6∗64 = 37824
	Maxpooling (ksize = 2)	64 × 576 × 576	0
Encoder block_2	DoubleConv_2	128 × 288 × 288	9∗(64 + 128)∗128 + 6∗128 = 221952
	Maxpooling (ksize = 2)	128 × 288 × 288	0
Encoder block_3	DoubleConv_3	256 × 144 × 144	9∗(128 + 256)∗256 + 6∗256 = 886272
	Maxpooling (ksize = 2)	256 × 144 × 144	0
Encoder block_4	DoubleConv_4	512 × 72 × 72	9∗(256 + 512)∗512 + 6∗512 = 3542016
	Maxpooling (ksize = 2)	512 × 72 × 72	0
Encoder block_5	DoubleConv_5	512 × 36 × 36	9∗(512 + 512)∗512 + 6∗512 = 4721664
	Maxpooling (ksize = 2)	512 × 36 × 36	0

Decoder block_1	Upsampling (bilinear)	512 × 72 × 72	0
	Concat	1024 × 72 × 72	0
	DoubleConv_6	256 × 72 × 72	9∗(1024 + 256)∗256 + 6∗256 = 2950656
Decoder block_2	Upsampling (bilinear)	256 × 144 × 144	0
	Concat	512 × 144 × 144	0
	DoubleConv_7	128 × 144 × 144	9∗(512 + 128)∗128 + 6∗128 = 738048
Decoder block_3	Upsampling (bilinear)	128 × 288 × 288	0
	Concat	256 × 288 × 288	0
	DoubleConv_8	64 × 288 × 288	9∗(256 + 64)∗64 + 6∗64 = 184704
Decoder block_4	Upsampling (bilinear)	64 × 576 × 576	0
	Concat	128 × 576 × 576	0
	DoubleConv_9	64 × 576 × 576	9∗(128 + 64)∗64 + 6∗64 = 110976

Output	Conv (ksize = 1)	1 × 576 × 576	1∗1∗64 + 1 = 65

**Table 2:** Segmentation results of improvements on DRIVE.

Channel	Se	Sp	ACC	AUC
Channel 1	0.8174	0.9768	0.9626	0.8971
Channel 2	0.8008	0.9741	0.9587	0.8875
Channel 3	0.8113	0.9748	0.9633	0.8931
3 channels of fusion	0.8745	0.9624	0.9546	0.9185

**Table 3:** Comparison of the proposed method with other methods on the DRIVE dataset.

Method	Se	Sp	ACC	AUC
Khan et al. (2016) [9]	0.7373	0.9670	0.9501	0.8522
Khan et al. (2016) [29]	0.780	0.972	0.952	0.876
Soomro et al. (2017) [11]	0.746	0.917	0.946	0.8315
Ngo and Han (2017) [12]	0.7464	0.9836	0.9533	0.8650
Biswal et al. (2017) [30]	0.71	0.97	0.95	0.84
Yan et al. (2018) [13]	0.7653	0.9818	0.9542	0.8736
Oliveira et al. (2018) [15]	0.8039	0.9804	0.9576	0.8922
Wang et al. (2019) [25]	0.7648	0.9817	0.9541	0.8733
Guo et al. (2019) [31]	0.7800	0.9806	0.9551	0.8803
Feng et al. (2019) [14]	0.7625	0.9809	0.9528	0.8717
Ribeiro et al. (2019) [32]	0.7880	0.9819	0.9569	0.8850
Dharmawan et al. (2019) [18]	0.8314	0.9726	—	0.902
Saroj et al. (2020) [5]	0.7307	0.9761	0.9544	0.8534
Dash and Senapati (2020) [33]	0.7403	0.9905	0.9661	0.8654
Biswas et al. (2020) [16]	0.7823	0.9814	0.9561	0.8819
Budak et al. (2020) [34]	0.7439	0.9900	0.9685	0.8670
2^nd^ human observer	0.7760	0.9724	0.9472	0.8742
Proposed method	0.8745	0.9624	0.9546	0.9185

**Table 4:** Comparison of the proposed method with other methods on the STARE dataset.

Method	Se	Sp	ACC	AUC
Khan et al. (2016) [9]	0.7359	0.9708	0.9502	0.8534
Khan et al. (2016) [35]	0.7728	0.9649	0.9518	0.8689
Khan et al. (2017) [36]	0.778	0.966	0.951	0.872
Soomro et al. (2017) [11]	0.748	0.922	0.948	0.835
Biswal et al. (2017) [30]	0.70	0.97	0.95	0.835
BahadarKhan et al. (2017) [37]	0.758	0.963	0.946	0.861
Yan et al. (2018) [13]	0.7581	0.9846	0.9612	0.8714
Oliveira et al. (2018) [15]	0.8315	0.9858	0.9694	0.9087
Wang et al. (2019) [25]	0.7523	0.9885	0.9640	0.8704
Guo et al. (2019) [31]	0.8201	0.9828	0.9660	0.9015
Feng et al. (2019) [14]	0.7709	0.9848	0.9633	0.8779
Dharmawan et al. (2019) [18]	0.7924	0.9827	—	0.8876
Saroj et al. (2020) [5]	0.7278	0.9724	0.9509	0.8501
Tamim et al. (2020) [38]	0.7806	0.9825	0.9632	0.8816
2^nd^ human observer	0.8952	0.9384	0.9349	0.9168
Proposed method	0.8903	0.9744	0.9699	0.9323

**Table 5:** Comparison of the proposed method with other methods on the CHASE_DB1 dataset.

Method	Se	Sp	ACC	AUC
Biswal et al. (2017) [30]	0.76	0.97	—	0.865
Yan et al. (2018) [13]	0.7633	0.9809	0.9610	0.8721
Oliveira et al. (2018) [15]	0.7779	0.9864	0.9653	0.8822
Wang et al. (2019) [25]	0.7730	0.9792	0.9603	0.8761
Guo et al. (2019) [31]	0.7888	0.9801	0.9627	0.8845
Soomro et al. (2019) [39]	0.8020	0.968	0.891	0.885
Tamim et al. (2020) [38]	0.7585	0.9846	0.9577	0.8716
Joshua et al. (2020) [40]	0.7796	0.9864	0.9722	0.8830
2^nd^ human observer	0.7686	0.9779	0.9560	0.8733
Proposed method	0.8916	0.9596	0.9561	0.9256

**Table 6:** Segmentation results of all test images of the three datasets.

Image	ACC	Se	Sp	AUC
DRIVE				
01_test	0.946	0.928	0.947	0.938
02_test	0.952	0.914	0.956	0.935
03_test	0.955	0.817	0.970	0.894
04_test	0.959	0.868	0.968	0.918
05_test	0.958	0.838	0.971	0.904
06_test	0.958	0.811	0.973	0.892
07_test	0.954	0.851	0.964	0.907
08_test	0.958	0.820	0.971	0.896
09_test	0.959	0.849	0.969	0.909
10_test	0.957	0.863	0.965	0.914
11_test	0.945	0.870	0.952	0.911
12_test	0.958	0.875	0.966	0.920
13_test	0.953	0.859	0.963	0.911
14_test	0.954	0.901	0.959	0.930
15_test	0.951	0.917	0.954	0.935
16_test	0.954	0.889	0.961	0.925
17_test	0.958	0.845	0.968	0.907
18_test	0.954	0.913	0.958	0.935
19_test	0.954	0.937	0.956	0.946
20_test	0.955	0.925	0.957	0.941
Avg.	0.955	0.875	0.962	0.918
STARE				
im0002	0.972	0.839	0.981	0.910
im0077	0.967	0.966	0.961	0.964
im0163	0.961	0.976	0.960	0.968
im0255	0.970	0.872	0.979	0.926
im0291	0.980	0.798	0.990	0.894
Avg.	0.970	0.890	0.974	0.932
CHASE_DB1				
11L	0.946	0.937	0.947	0.942
11R	0.942	0.950	0.942	0.946
12L	0.953	0.878	0.959	0.919
12R	0.958	0.872	0.965	0.918
13L	0.958	0.884	0.963	0.923
13R	0.956	0.850	0.963	0.907
14L	0.970	0.895	0.968	0.931
14R	0.966	0.867	0.971	0.919
Avg.	0.956	0.892	0.960	0.926

**Table 7:** MCC and CAL metrics of existing techniques on the three datasets.

Method	DRIVE		STARE		CHASE_DB1	
	MCC	CAL	MCC	CAL	MCC	CAL
Azzopardi et al. (2015) [41]	0.719	0.721	0.698	0.709	0.656	0.608
Orlando et al. (2016) [42]	0.740	0.675	0.726	0.665	0.689	0.571
Dharmawan et al. (2017) [18]	07991	0.8834	0.7959	0.8181	—	—
Yang et al. (2018) [43]	0.725	—	0.662	—	—	—
Strisciuglio et al. (2019) [44]	0.729	0.728	0.698	0.709	0.663	0.620
Khan et al. (2020) [45]	0.739	0.696	0.707	0.566	0.629	0.547
2^nd^ human observer	0.770	0.771	0.741	0.622	0.626	0.722
Proposed method	0.756	0.796	0.796	0.837	0.566	0.733

**Table 8:** Network comparison of the proposed method with other methods based on U-Net.

Method	Input size	Epoch	Training images	Parameters
Ronneberger et al. (2015) [24]	572∗572∗1	N/A	N/A	28.94 M
Yan et al. (2018) [13]	128∗128∗1	Over 30	26052 (4)	30.96 M
Jiang et al. (2018) [19]	500∗500∗3	30	42421 (4)	58.31 M
Soomro et al. (2019) [39]	Original image	N/A	3959 (4)	4.71 M
Joshua et al. (2020) [40]	512∗512∗3	50	113 (4)	0.64 M
Zhang et al. (2020) [17]	256∗256∗1	120	116 (3)	3.86 M
Budak et al. (2020) [34]	48∗48∗3	Over 30	Over 45840 (2)	0.97 M
Proposed method	576∗576∗1	30	220 (3)	13.39 M

*x* (*y*) means *x* training images of *y* datasets.

## Data Availability

The three public open-source datasets used to support this study are available at http://www.isi.uu.nl/Research/Databases/DRIVE/, http://cecas.clemson.edu/~ahoover/stare/, and https://blogs.kingston.ac.uk/retinal/chasedb1/.

## Supplementary Materials

Supplementary Materials Table 1: channel 1 results of DRIVE test images. Table 2: channel 1 results of STARE test images. Table 3: channel 1 results of CHASE_DB1 test images.
