# Supplementary material for: Multichannel Retinal Blood Vessel Segmentation Based on the Combination of Matched Filter and U-Net Network
Source: Biomed Res Int. 2021 May 25;2021:5561125. doi: 10.1155/2021/5561125 (PMC8172291; doi:10.1155/2021/5561125)
Supplement: Supplementary Materials — Table 1: channel 1 results of DRIVE test images. Table 2: channel 1 results of STARE test images. Table 3: channel 1 results of CHASE_DB1 test images. [file 5561125.f1.docx]

See Table 1-3.

Table 1: Channel 1 results of DRIVE test images

| **Image** | **Proposed method** | | | | | | **Without CLAHE** | | | | | |
| --- | --- | --- | --- | --- | --- | --- | --- | --- | --- | --- | --- | --- |
|  | **Acc** | **Se** | **Sp** | **AUC** | **MCC** | **CAL** | **Acc** | **Se** | **Sp** | **AUC** | **MCC** | **CAL** |
| 01_test | 0.957 | 0.860 | 0.967 | 0.913 | 0.762 | 0.770 | 0.958 | 0.848 | 0.968 | 0.908 | 0.761 | 0.757 |
| 02_test | 0.962 | 0.857 | 0.974 | 0.916 | **0.802** | 0.758 | 0.963 | 0.843 | 0.977 | 0.910 | **0.804** | 0.756 |
| 03_test | 0.955 | 0.731 | 0.980 | 0.856 | 0.741 | 0.624 | 0.954 | 0.667 | 0.986 | 0.826 | 0.726 | 0.546 |
| 04_test | 0.962 | 0.746 | 0.984 | 0.865 | 0.763 | 0.551 | 0.957 | 0.580 | **0.995** | 0.787 | 0.712 | 0.359 |
| 05_test | 0.963 | 0.759 | 0.984 | 0.871 | 0.773 | 0.635 | 0.963 | 0.758 | 0.984 | 0.871 | 0.774 | 0.635 |
| 06_test | 0.959 | 0.714 | **0.985** | 0.850 | 0.752 | 0.576 | 0.959 | 0.716 | 0.985 | 0.851 | 0.755 | 0.581 |
| 07_test | 0.961 | 0.781 | 0.979 | 0.880 | 0.762 | 0.635 | 0.962 | 0.753 | 0.983 | 0.868 | 0.761 | 0.604 |
| 08_test | 0.961 | 0.726 | 0.983 | 0.855 | 0.741 | 0.610 | 0.960 | 0.673 | 0.987 | 0.830 | 0.725 | 0.541 |
| 09_test | 0.961 | 0.774 | 0.978 | 0.876 | 0.743 | 0.618 | 0.965 | 0.754 | 0.984 | 0.869 | 0.759 | 0.636 |
| 10_test | 0.963 | 0.784 | 0.979 | 0.882 | 0.757 | 0.665 | 0.963 | 0.788 | 0.979 | 0.884 | 0.761 | 0.648 |
| 11_test | 0.957 | 0.793 | 0.973 | 0.883 | 0.743 | 0.617 | 0.960 | 0.732 | 0.982 | 0.857 | 0.745 | 0.550 |
| 12_test | 0.961 | 0.743 | 0.982 | 0.862 | 0.747 | 0.624 | 0.963 | 0.739 | 0.984 | 0.861 | 0.754 | 0.636 |
| 13_test | 0.958 | 0.791 | 0.976 | 0.884 | 0.764 | 0.675 | 0.959 | 0.770 | 0.979 | 0.875 | 0.763 | 0.638 |
| 14_test | 0.962 | 0.844 | 0.972 | 0.908 | 0.762 | 0.722 | 0.964 | 0.820 | 0.977 | 0.898 | 0.768 | 0.717 |
| 15_test | **0.965** | 0.837 | 0.975 | 0.906 | 0.756 | 0.709 | **0.967** | 0.823 | 0.978 | 0.900 | 0.763 | 0.712 |
| 16_test | 0.960 | 0.810 | 0.975 | 0.893 | 0.764 | 0.720 | 0.959 | 0.800 | 0.975 | 0.888 | 0.758 | 0.700 |
| 17_test | 0.959 | 0.746 | 0.979 | 0.863 | 0.733 | 0.623 | 0.961 | 0.748 | 0.980 | 0.864 | 0.742 | 0.639 |
| 18_test | 0.959 | 0.850 | 0.969 | 0.909 | 0.749 | 0.730 | 0.961 | 0.831 | 0.972 | 0.901 | 0.751 | 0.725 |
| 19_test | 0.964 | **0.886** | 0.971 | **0.928** | 0.787 | **0.782** | 0.965 | **0.876** | 0.973 | **0.924** | 0.790 | **0.779** |
| 20_test | 0.964 | 0.837 | 0.974 | 0.906 | 0.756 | 0.751 | 0.963 | 0.784 | 0.977 | 0.881 | 0.737 | 0.680 |
| Avg. | 0.961 | 0.793 | 0.977 | 0.885 | 0.758 | 0.670 | 0.961 | 0.765 | 0.980 | 0.873 | 0.755 | 0.642 |

Table 2: Channel 1 results of STARE test images

| **Image** | **Proposed method** | | | | | | **Without CLAHE** | | | | | |
| --- | --- | --- | --- | --- | --- | --- | --- | --- | --- | --- | --- | --- |
|  | **Acc** | **Se** | **Sp** | **AUC** | **MCC** | **CAL** | **Acc** | **Se** | **Sp** | **AUC** | **MCC** | **CAL** |
| im0002 | 0.976 | 0.795 | 0.989 | 0.892 | 0.802 | 0.721 | 0.975 | 0.770 | 0.990 | 0.880 | 0.794 | 0.687 |
| im0077 | 0.971 | 0.923 | 0.975 | 0.949 | 0.825 | 0.861 | 0.972 | 0.912 | 0.977 | 0.945 | 0.827 | 0.860 |
| im0163 | 0.968 | 0.947 | 0.970 | 0.959 | 0.815 | 0.866 | 0.973 | 0.926 | 0.977 | 0.952 | 0.833 | 0.875 |
| im0255 | 0.972 | 0.781 | 0.991 | 0.886 | 0.820 | 0.758 | 0.971 | 0.755 | 0.992 | 0.874 | 0.810 | 0.731 |
| im0291 | 0.977 | 0.693 | 0.992 | 0.842 | 0.740 | 0.561 | 0.969 | 0.474 | 0.996 | 0.735 | 0.623 | 0.289 |
| Avg. | 0.974 | 0.846 | 0.984 | 0.915 | 0.802 | 0.797 | 0.959 | 0.622 | 0.983 | 0.802 | 0.647 | 0.489 |

Table 3: Channel 1 results of CHASE_DB1 test images

| **Image** | **Proposed method** | | | | | | **Without CLAHE** | | | | | |
| --- | --- | --- | --- | --- | --- | --- | --- | --- | --- | --- | --- | --- |
|  | **Acc** | **Se** | **Sp** | **AUC** | **MCC** | **CAL** | **Acc** | **Se** | **Sp** | **AUC** | **MCC** | **CAL** |
| 11L | 0.943 | 0.882 | 0.945 | **0.914** | 0.548 | 0.557 | 0.947 | 0.853 | 0.950 | 0.901 | 0.549 | 0.561 |
| 11R | 0.938 | **0.901** | 0.939 | 0.920 | 0.540 | 0.540 | 0.943 | **0.896** | 0.945 | **0.920** | 0.557 | 0.583 |
| 12L | 0.943 | 0.808 | 0.949 | 0.878 | 0.567 | 0.632 | 0.945 | 0.788 | 0.953 | 0.870 | 0.568 | 0.625 |
| 12R | 0.947 | 0.805 | 0.954 | 0.879 | 0.588 | 0.674 | 0.944 | 0.796 | 0.957 | 0.876 | 0.595 | 0.676 |
| 13L | 0.950 | 0.788 | 0.956 | 0.872 | 0.559 | 0.598 | 0.943 | 0.743 | 0.961 | 0.852 | 0.553 | 0.597 |
| 13R | 0.951 | 0.749 | 0.960 | 0.854 | 0.550 | 0.609 | 0.950 | 0.725 | 0.959 | 0.842 | 0.534 | 0.572 |
| 14L | 0.951 | 0.836 | 0.956 | 0.896 | **0.605** | **0.690** | 0.941 | 0.833 | 0.957 | 0.895 | **0.604** | **0.690** |
| 14R | **0.959** | 0.797 | **0.965** | 0.881 | 0.600 | 0.664 | **0.959** | 0.768 | **0.967** | 0.867 | 0.588 | 0.638 |
| Avg. | 0.948 | 0.821 | 0.953 | 0.887 | 0.570 | 0.621 | 0.947 | 0.800 | 0.956 | 0.878 | 0.568 | 0.618 |
